# Delayed *Propionibacterium acnes* surgical site infections occur only in the presence of an implant

**DOI:** 10.1038/srep32758

**Published:** 2016-09-12

**Authors:** Yuta Shiono, Ken Ishii, Shigenori Nagai, Hiroaki Kakinuma, Aya Sasaki, Haruki Funao, Tetsuya Kuramoto, Kenji Yoshioka, Hiroko Ishihama, Norihiro Isogai, Kenichiro Takeshima, Takashi Tsuji, Yasunori Okada, Shigeo Koyasu, Masaya Nakamura, Yoshiaki Toyama, Mamoru Aizawa, Morio Matsumoto

**Affiliations:** 1 Department of Orthopaedic Surgery, Keio University School of Medicine, 35 Shinanomachi, Shinjuku-ku, Tokyo, 160-8582, Japan; 2 Department of Microbiology and Immunology, Keio University School of Medicine, 35 Shinanomachi, Shinjuku-ku, Tokyo, 160-8582, Japan; 3 Core Research for Evolutional Science and Technology (CREST), Japan Science and Technology Agency (JST), Tokyo, Japan; 4 Department of Molecular Immunology, Graduate School of Medical and Dental Sciences, Tokyo Medical and Dental University, Tokyo, Japan; 5 Department of Applied Chemistry, School of Science and Technology, Meiji University, 1-1-1 Higashimita, Tama-ku, Kawasaki, Kanagawa, 214-8571, Japan; 6 Department of Pathology, Keio University School of Medicine, 35 Shinanomachi, Shinjuku-ku, Tokyo, 160-8582, Japan; 7 Laboratory for Immune Cell Systems, RIKEN Center for Integrative Medical Sciences (IMS), Yokohama, Kanagawa, Japan

## Abstract

Whether *Propionibacterium acnes* (*P. acnes*) causes surgical-site infections (SSI) after orthopedic surgery is controversial. We previously reported that we frequently find *P. acnes* in intraoperative specimens, yet none of the patients have clinically apparent infections. Here, we tracked *P. acnes* for 6 months in a mouse osteomyelitis model. We inoculated *P. acnes* with an implant into the mouse femur in the implant group; the control group was treated with the bacteria but no implant. We then observed over a 6-month period using optical imaging system. During the first 2 weeks, bacterial signals were detected in the femur in the both groups. The bacterial signal completely disappeared in the control group within 28 days. Interestingly, in the implant group, bacterial signals were still present 6 months after inoculation. Histological and scanning electron-microscope analyses confirmed that *P. acnes* was absent from the control group 6 months after inoculation, but in the implant group, the bacteria had survived in a biofilm around the implant. PCR analysis also identified *P. acnes* in the purulent effusion from the infected femurs in the implant group. To our knowledge, this is the first report showing that *P. acnes* causes SSI only in the presence of an implant.

Surgical site infections (SSI) pose serious postoperative complications. Various pathogens can cause SSI associated with implanted medical devices. Implant-associated infections (IAI) are typically caused by microorganisms growing in structures known as biofilms. In the orthopedic field, SSI are commonly caused by aerobic bacteria, such as *Staphylococcus aureus*, *S. epidermidis*, *and Pseudomonas aeruginosa*^1^,^2^,^3^,^4^. *Propionibacterium acnes* (*P. acnes*), an anaerobe, is also reported to cause SSI and IAI following orthopedic surgeries. In contrast, recent studies have shown that the prevalence is probably underestimated according to several points: slow and low growing bacteria, anaerobic conditions not always well-performed, ability of the lab and potential interaction with other bacteria especially *S. epidermidis* with bacteriocins^5^,^6^. Richards and Emara^7^ examined 489 patients who underwent surgery for idiopathic scoliosis, of whom 23 developed delayed infections. When the instrumentation was removed, specimens were found to be strongly positive for *P. acnes* in 12 (53%) of the 23 patients. Although *P. acnes* is considered to have low pathogenic potential, Bayston^8^ reported that it forms biofilms, which are difficult to remove and treat with antibiotics. Achermann *et al*.^9^ reveled that treatment for SSI caused by *P. acnes* requires a combination of surgery and a prolonged antibiotic treatment regimen to successfully eliminate the remaining bacteria. Most authors suggest a course of 3 to 6 months of antibiotic treatment, including 2 to 6 weeks of intravenous treatment with a beta-lactam. The role of rifampin in *P. acnes* has been studied^10^: those authors reported *in vivo* data and data from an experimental animal model showing a good efficacy of rifampin alone and in combination with vancomycin, daptomycin, or levofloxacin. After retrieving infected spinal implants and sampling the bacterial biofilm on the implant surface using the vortexing-bath sonication technique, Sampedro *et al*.^11^ concluded that *P. acnes* might be the most frequently involved in IAIs. It is also recognized that *P. acnes* is an important pathogen in cerebrovascular infections^12^, fibrosis of breast implants^13^, and infections of cardiovascular devices^14^.

*P. acnes* is found ubiquitously on the skin and in other areas of the body, including the upper respiratory and gastrointestinal tracts^15^. Levy *et al*.^16^ identified *P. acnes* via conventional aerobic and anaerobic culturing techniques in 41.8% (23 of 55) of consecutive patients who had no outward signs of infection and who were undergoing primary shoulder arthroplasty. We previously reported finding *P. acnes* in intraoperative specimens from 11 of 80 patients (14%) who underwent scoliosis surgery, although none of the patients had symptoms of infection^17^. In these studies, possible reasons for the infection not becoming established included 1) a false-positive detection of *P. acnes* due to contamination; 2) insufficient numbers of bacteria to establish the infection; or 3) the inability of *P. acnes* to grow in an aerobic environment *in vivo*. Thus, these clinical studies did not clarify whether *P. acnes* could survive and cause IAI.

Recent developments in small animal optical imaging, including fluorescence imaging (FRI) and bioluminescence imaging (BLI), have significantly contributed to basic research. These tools use bioluminescent and fluorescent reporters and/or probes to provide real-time, non-invasive sequential monitoring of cell growth and gene expression *in vivo*, enabling us to monitor transplanted cells in live animals in real time^18^,^19^,^20^,^21^. Inoculated bacteria that emit a constant fluorescent or bioluminescent signal can be detected through the tissues of a live animal using an ultrasensitive, cooled, charge-coupled device (CCD) camera. This approach has proved to be useful in studies in the fields of oncology^22^, endocrine disruptors^23^, metabolism^24^, regenerative medicine^18^,^19^, immunology^25^, and infections^20^,^21^,^26^,^27^,^28^. Several fluorescent proteins and dyes, including green fluorescent protein, red fluorescent protein, and cyanine dyes (Cy2, Cy3, and Cy5) can be visualized through animal tissues. In general, high-excitation fluorescent dyes, such as near-infrared (NIR) fluorescent probes, provide adequate signal, deeper tissue penetration, and lower background noise than those previously possible.

Transfection of the luciferase gene (*Luc*) into bacteria is usually performed by competent-cell or protoplast transformation. However, the rate of successful transformation is typically low because *Luc* is quite large (approximately 18.4 kb)^29^. Some investigators have reported that transferring plasmid DNA via electroporation greatly facilitates the application of recombinant DNA methodology and transposon technology in gram-positive bacteria^30^. However, the rate of transformation of plasmid DNA into gram-positive bacteria is often low. In the present study, we successfully visualized and tracked *P. acnes* labeled with a bacterial membrane-binding NIR-fluorescent probe to determine the time course of *P. acnes* infection *in vivo.*

## Results

### High adherence ability of *P. acnes* on the titanium alloy

**Figure 1:**
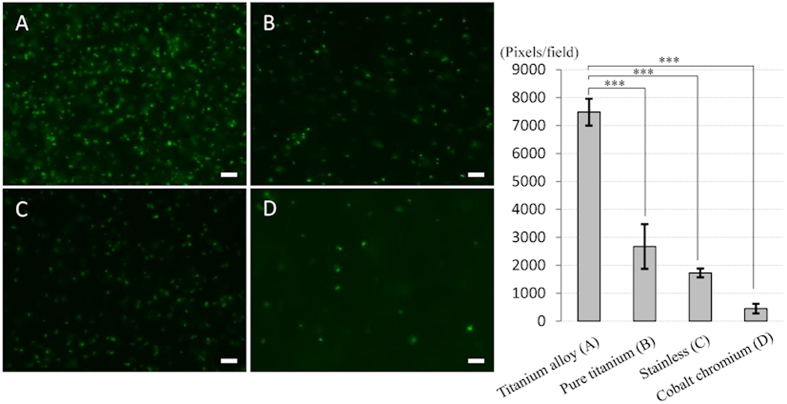
Bacterial adhesion and quantification of biofilm formation on various metals *in vitro.* Four different metal plates (5-mm in diameter) were immersed into *P. acnes*-cultured medium (1 × 10^6^ colony forming units (CFU)/ml) at 37 °C for 24 h. *P. acnes* was labeled with LIVE/DEAD^®^ BacLight after washing with PBS. Titanium alloy (**A**), which is frequently used clinically, had significantly higher bacterial adhesion among 4 different metals. Bars = 10 μm. The average densities of biofilm on 4 metals were high in the order of titanium alloy (**A**) > pure titanium (**B**) > stainless (**C**) > cobalt–chromium (**D**) (p < 0.001).

*In vitro* bacterial adherence analyses revealed bacterial adherence and biofilm on the surface of all metals. Interestingly, the ability of *P. acnes* to form a biofilm varied by material; the biofilm on the titanium alloy was significantly denser than that on the other metals, including pure titanium, stainless, or cobalt–chromium alloy (Fig. 1). The average densities of biofilm on 4 metals were high in the order of titanium alloy > pure titanium > stainless > cobalt–chromium (p < 0.001).

### Bacterial Probe Specifically Binds to *P. acnes in vitro* and *in vivo*

**Figure 2:**
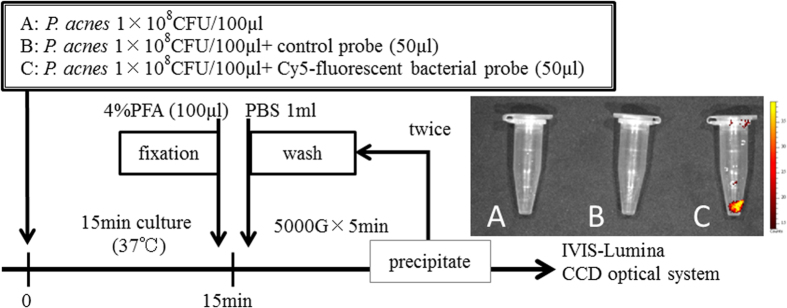
*P. acnes* visualization by a NIR-fluorescent bacterial probe *in vitro.* Three small Eppendorf tubes containing 1 × 10^8^ colony forming units (CFU) of *P. acnes* in 100 μl of medium were prepared after incubating each tube with 50 μl of medium (**A**), a NIR-fluorescent control probe (**B**), or a NIR-fluorescent bacterial detection probe (**C**) at 37.0 °C under anaerobic conditions for 15 min. The cell suspension was fixed with 4% paraformaldehyde (PFA), washed twice with phosphate-buffered saline (PBS), and centrifuged at 5000 g for 5 min to collect precipitated bacteria and to decant the supernatant. Next, NIR-fluorescent signals from the bacterial probe bound to the bacterial cell membrane were detected using an IVIS^®^-Lumina CCD optical system. No fluorescent signals were detected from untreated *P. acnes* (**A**) or *P. acnes* incubated with a NIR-fluorescent control probe (**B**). In contrast, strong signal was detected from *P. acnes* incubated with the NIR-fluorescent bacterial probe (**C**).

**Figure 3:**
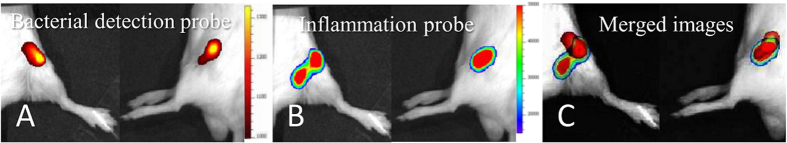
Visualization of the inflammatory area in *P. acnes* osteomyelitis. A NIR-fluorescent bacterial detection probe and a chemiluminescent inflammation probe were injected into each mouse. The *P. acnes* growth area (**A**) and inflammatory area (**B**) were observed by fluorescence imaging (FRI) and bioluminescence imaging (BLI), respectively, in each mouse. The merged images revealed that the luminescent inflammation area covered most of the bacterial fluorescent signal area (**C**). The chemiluminescent inflammation probe specifically bound to neutrophils at the inflammation area in the *P. acnes* osteomyelitis.

**Figure 4:**
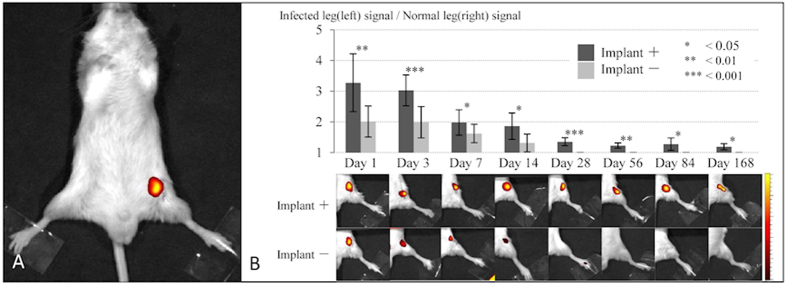
Time course of optical imaging of *P. acnes* osteomyelitis with or without implant. NIR-fluoresence signals from the bacterial probe were successfully detected at the infected area (**A**) signals were sequentially measured on days 1, 3, 7, 14, 28, 56, 84 (3 months), and 168 (6 months) after the inoculation in the implant group (implant +) (N = 12) and the control group (implant −) (N = 6) (**B**). In the control group, clear signals were detected only for the first 14 days and the signal had completely disappeared within 28 days, indicating that the inoculated *P. acnes* could not survive for >28 days in this model. In contrast, signals in the implant group were detected 6 months after the *P. acnes* inoculation. At each time point, fluorescence ratios calculated from bacterial PIs in the implant group were significantly higher than those in the control group (p < 0.05 each). Means ± standard deviation (SD) are shown.

In our *in vitro* analysis using the IVIS^®^ optical imaging system, no fluorescent signals were detected from the untreated *P. acnes* or from *P. acnes* incubated with the NIR-fluorescent control probe (Fig. 2A,B). In contrast, a strong signal was detected from *P. acnes* incubated with the NIR-fluorescent bacterial probe (Fig. 2C). *In vivo* optical imaging successfully detected the bacterial fluorescence signal in the femur (Figs 3A and 4A). These results indicated that the bacterial probe specifically bound to the *P. acnes* cell membrane *in vitro* and *in vivo.*

### *P. acnes* infection causes an inflammatory response

We used both bacterial and inflammation probes to observe the growth area of *P. acnes* and the area of the inflamed region simultaneously in individual animals. We successfully detected the luminescent signal around the bacterial signal in mouse femurs 7 days after surgery and immediately after injecting the inflammation probe (Fig. 3B). Interestingly, merged images showed that the luminescent inflammation area covered most of the bacterial fluorescent signal area (Fig. 3C). Because the inflammation probe specifically binds to neutrophils at the inflammation area, these results indicated that *P. acnes* causes bacterial inflammation and induces neutrophils. To our knowledge, this is the first report visualizing a *P. acnes* bacterial infection and the resulting inflammation in living animals.

### *P. acnes* caused delayed SSI only in the presence of an implant

Bacterial signals in both the implant group (N = 12) and in the control group (N = 6) were captured by an IVIS^®^ optical imaging system at various time points over the course of 6 months (Fig. 4B). In both groups, bacterial signals were clearly identified in the femur for the first 3 days. In the control group, the signal greatly decreased at 14 days and completely disappeared within 28 days. Thus, the inoculated *P. acnes* did not survive >28 days in this model. Interestingly, signals were detected in the implant group 6 months after inoculation. For the first 14 days, the fluorescence ratios calculated from bacterial PIs in the implant group were approximately 1.5 times greater than those in the control group at the various time points (implant group *vs* control group: day 1, 3.28 ± 0.94 *vs* 2.01 ± 0.50; day 3, 3.03 ± 0.50 *vs* 1.99 ± 0.51; and day 7; 1.98 ± 0.41 *vs* 1.62 ± 0.30). The fluorescence ratios on days 1, 3, 7, 14, 28, 56, 84, and 168 were significantly higher in the implant group than in the control group (p < 0.05 each) (Fig. 4B).

### Histological analysis

**Figure 5:**
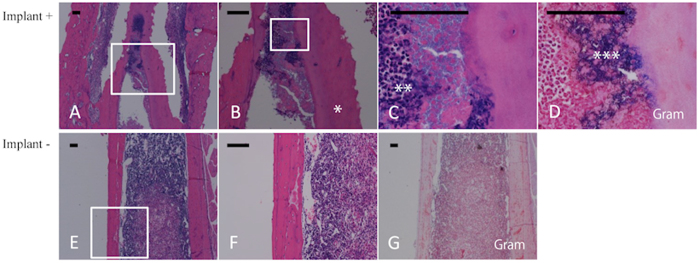
Histological analysis of implant-group and control-group femurs. Longitudinal sections of femurs obtained 28 days after bacterial inoculation from the implant group (**A–D**) and the control group (**E–G**) were subjected to hematoxylin–eosin staining (**A–C**, **E**,**F**) and Gram staining (**D**,**G**). (**B**,**C**,**D**,**F)** show magnified views of the white-boxed areas in (**A**,**B**,**E)**, respectively. Gram-positive bacterial colonies were detected in the medullary cavity of the infected mouse femur (**D**) along with a marked infiltration of neutrophils (**A–C**). New bone formation and trabecular bone resorption by osteoclasts were also observed (**A**,**B**). In the control group, *P. acnes* had disappeared and the infection had subsided (**E–G**). *Gram-positive bacteria, **Necrotic neutrophils, ***Involucrum, Bars = 100 μm.

On day 28, we observed sequestrum, new bone formation, fibrosis, and other manifestations of chronic osteomyelitis in the implant group (Fig. 5A–D). Gram-positive bacterial colonies were present in the medullary cavity of the infected mouse femurs (Fig. 5D) along with a marked infiltration of neutrophils (Fig. 5A–C). We also observed new bone formation and trabecular bone resorption by osteoclasts (Fig. 5A,B). However, in the control group, *P. acnes* disappeared and the infection subsided (Fig. 5E–G).

### *P. acnes* produced a biofilm on the implant surface

**Figure 6:**
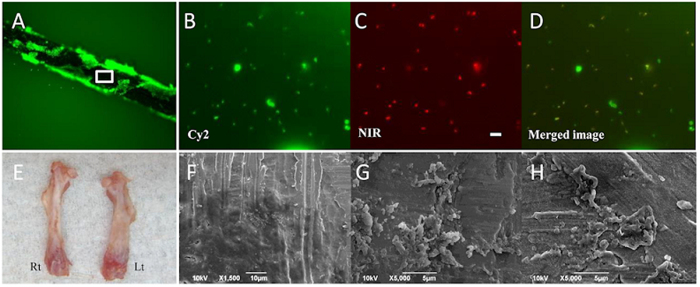
*Ex vivo* and scanning electron microscopy (SEM) analyses. In the implant group, many *P. acnes* labeled with Cy2 LIVE/DEAD^®^ BacLight were observed on the implant surface (**A**). In the biofilm, bacteria labeled with Cy2 (**B**) also expressed the NIR fluorescent dye (**C**), suggesting that the NIR fluorescent signals observed by optical imaging originated from the inoculated *P. acnes* labeled with the bacterial probe. (**D**) shows the merged image. Bars = 5 μm. Six months after the inoculation, the infected left femur in the implant group was typically shorter than the normal femur on the right side and had a curved deformation with cortical thickening (**E**). SEM findings from the implant surface 6 months after the inoculation revealed many *P. acnes* and biofilm (**F**–**H**).

In the implant group, many *P. acnes* labeled with Cy2 LIVE/DEAD^®^ BacLight were observed on the implant surface (Fig. 6A). In this biofilm, the bacteria labeled with Cy2 LIVE/DEAD^®^ BacLight (Fig. 6B) also expressed the NIR fluorescent dye (Fig. 6C,D), suggesting that the NIR fluorescent signals observed by optical imaging originated from the inoculated *P. acnes* detected by the bacterial probe. Three months after inoculation, *P. acnes* survived on the implant and moved aggressively inside the biofilm. Interestingly, 6 months after the inoculation, the infected left femur in the implant group was typically shorter than the right (normal) femur and had a curved deformation with cortical thickening; this was not observed in the control group (Fig. 6E). Scanning electron microscope (SEM) findings of the implant surface 6 months after surgery revealed many *P. acnes* and biofilms on the implant surface (Fig. 6F–H).

### Genetic analysis

**Figure 7:**
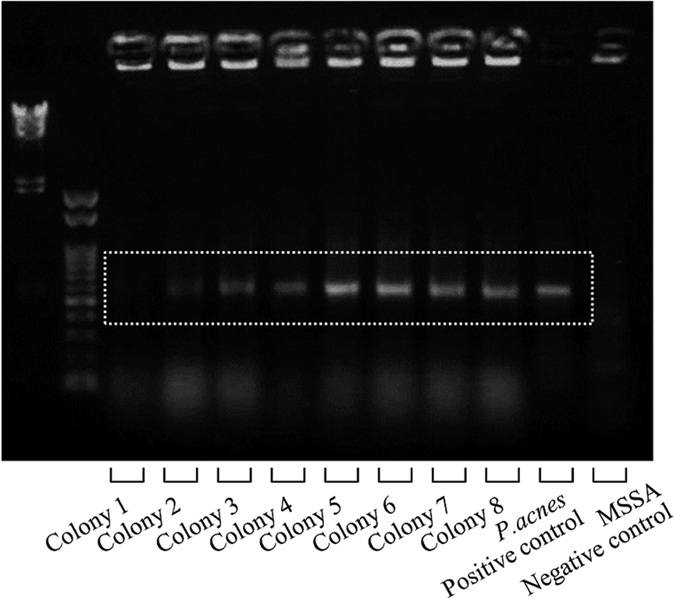
PCR-confirmed genetics of the *P. acnes* obtained from femur specimens 6 months after inoculation. Six months after inoculation, purulent effusions from infected femurs were cultured under anaerobic conditions for 2 weeks; 8 colonies were obtained and were confirmed to be exactly the same strain of *P. acnes* (ATCC 51277, Strain Designations VPI 9) by PCR analysis, showing that *P. acnes* could survive for 6 months only in the presence of an implant.

In the implant and control groups, effusions from femurs were cultured under anaerobic conditions for 2 weeks, resulting in the detection of eight colonies only in the implant group. Each colony was analyzed by PCR. All of the bacteria from the eight colonies were exactly the same strain of *P. acnes* (ATCC 51277, Strain Designations VPI 9) (Fig. 7). These results show that *P. acnes* could survive only in the presence of an implant for 6 months without contamination and retained the capacity for growth *in vivo*. In other words, *P. acnes* observed in the biofilm on the implant surface might produce anaerobic conditions to survive.

## Discussion

*P. acnes* is generally ubiquitous in the deep layers of the skin, in the upper respiratory and gastrointestinal tracts, and in eye mucosa^15^,^31^. When the mucous membranes and skin are incised using a scalpel or penetrated using a needle, the exposed tissues can be contaminated with endogenous flora^17^,^32^. Several studies have shown that *P. acnes* causes SSI^33^,^34^,^35^,^36^ and late infections^3^,^37^,^38^, whereas other reports have suggested that the high rates of *P. acnes*-positive cultures is due to sample contamination^39^. In addition, *P. acnes* is generally believed to have a low pathogenic potential^17^. Therefore, whether *P. acnes* causes delayed SSI is controversial. Sampedro *et al*.^11^ removed spinal implants from 22 patients with SSI, cultured the peri-implant tissues, and sonicated the fluid from the retrieved implants; they detected *P. acnes* in 9 (41%) of the 22 patients. In contrast, Levy *et al*.^16^ identified *P. acnes* using conventional aerobic and anaerobic culturing techniques in 41.8% (23 of 55) of consecutive patients who had no outward signs of infection and were undergoing primary shoulder arthroplasty. We previously reported detecting *P. acnes* in intraoperative specimens from 11 of 80 patients (14%) who underwent scoliosis surgery; none of the patients had symptoms of infection^17^. Thus, it was considered that *P. acnes* could be discounted as a pathogen in SSI and that high positive rates of *P. acnes* probably reflected false-positive results. Therefore, little was elucidated about the time course of *P. acnes* infections.

In the present study, we visualized and tracked the growth of living *P. acnes* using an optical imaging system. *Ex vivo* analysis of removed implants showed Cy2 LIVE/DEAD^®^-labeled bacteria aggressively moving on the implant surface and expressing NIR fluorescent dye, suggesting that the NIR fluorescent signal observed by optical imaging originated from the inoculated *P. acnes* detected by the bacterial detection probe. Using an inflammation probe that specifically binds to neutrophils, we could simultaneously visualize the growth of *P. acnes* and the inflamed region. Analysis of the time course *in vivo* showed that the inoculated *P. acnes* could not survive in the femur for >28 days without an implant. Interestingly, we found that *P. acnes* was able to survive for >6 months when the IAI was associated with a titanium alloy implant, suggesting that the presence of the implant was essential for the survival of the bacteria and the development of IAI. To the best of our knowledge, this is the first report demonstrating that *P. acnes* causes SSI only in the presence of an implant.

It was previously reported that bacterial biofilm is an anaerobic environment^40^. The formation of a bacterial biofilm is of major concern with indwelling medical devices because the biofilm provides bacteria with considerable resistance to host defenses and antimicrobial agents^40^,^41^. Single bacterial cells can be easily eliminated by macrophages or neutrophils in a normal, healthy host; however, bacterial colonies enclosed in a self-produced polymeric sugar matrix are far more resistant to oxygen radicals and phagocytosis^40^. Indeed, it was shown in animal models that *S. epidermidis* strains that produce extracellular polysaccharides (EPSs) are more virulent than EPS-negative *S. epidermidis* strains^42^,^43^. The self-produced EPS that forms a matrix around the bacteria may impair the penetration of antimicrobial agents. Once a biofilm has formed, the low metabolic rate of the bacteria may also limit the effectiveness of antibiotics, which require active cell division and active metabolism^44^. Although *P. acnes* has low pathogenic potential, in the present study, the bacteria produced EPSs to form a biofilm on the implant and to survive the host’s immune system. Furthermore, *P. acnes* may be able to survive for an extended period by forming an anaerobic microenvironment within the biofilm. In contrast, Furustrand Tafin *et al*.^10^ have demonstrated that a high inoculation (1 × 10^9 ^CFU/ cage) could create an infection with a biofilm, but not a low inoculation (5 × 10^7 ^CFU/ cage) using tissue cage infection model. Therefore, the persistence of infection with biofilm formation may be not solely related to presence of implant, but also the kind and the amount of microorganism, its specific adhesion molecules, implant characteristics, and so on.

In the present study, we showed that *P. acnes* SSI can be reliably established around a titanium alloy implant. *In vitro* bacterial adherence experiment examined the ability of *P. acnes* to form biofilms *in vitro* on four different metals, including stainless, titanium alloy, pure titanium, and cobalt–chromium alloy, that are frequently used in orthopedic surgery^45^. Interestingly, the ability of *P. acnes* to form a biofilm varied by material; the biofilm on the titanium alloy was significantly denser than that on the other metals (Fig. 1). If the biofilm cannot form on the metal, anaerobic bacteria, such as *P. acnes*, may not be able to survive *in vivo*, which might prevent IAI. In other words, modifying the implant surface with an antibacterial coating^46^,^47^, printing technologies^48^, or nanotechnology^49^ may effectively prevent SSI.

In conclusion, we showed that *P. acnes* causes delayed SSI in a mouse osteomyelitis model and that the presence of an implant is essential for the development of SSI.

## Materials and Methods

### *P. acnes* bacterial strain and culture conditions

We obtained *P. acnes* (ATCC No. 51277, Strain Designations VPI 9) from American Type Culture Collection (ATCC, Manassas, VA, USA) and cultured it in Reinforced Clostridial Medium (ATCC medium 1053) at 37.0 °C under anaerobic conditions with 150 rpm agitation for 16 h. The bacterial samples were frozen and stored at −80 °C. The samples were thawed at 4 °C for 1 h prior to each experiment.

### Adherence ability of *P. acnes* on the various materials

In a preliminary experiment, we examined the adherence ability of *P. acnes* to form biofilms *in vitro* on four different metals that are frequently used in orthopedic surgery. We prepared a titanium alloy (Ti-6Al-4VELI), pure titanium, stainless (SUS316L), and a cobalt–chromium alloy (Co–Cr–Mo). Each metal plate (5-mm diameter) was incubated in a Reinforced Clostridial Medium containing *P. acnes* [1 × 10^6^ colony-forming units (CFU)/μl] at 37.0 °C under anaerobic conditions for 24 h. Bacterial adherence to the metallic plates was observed using LIVE/DEAD Baclight fluorescence staining (Life Technologies Japan, Tokyo, Japan)^45^. Five fields in the biofilms were selected. The total numbers of pixels in the fluorescence staining area were measured in five fields from six sections using SigmaScan Pro Image software (SPSS Inc., Chicago, IL, USA).

### Optical imaging

Optical imaging was performed with a Caliper LS-IVIS^®^ Lumina cooled CCD optical macroscopic imaging system (Summit Pharmaceuticals International Co., Tokyo, Japan). To detect the inoculated *P. acnes*, a NIR-fluorescent bacterial probe, the XenoLight Bacterial Detection Probe 750 (Summit Pharmaceuticals International Co., Tokyo, Japan), was used. This bacterial probe shows a peak excitation at 750 nm and peak emission at 780 nm; the ideal spectrum filter sets would be Excitation 745 nm/ Emission 800 nm. The probe specifically targets anionic phospholipids in the bacterial cell membrane and targets both gram-positive and gram-negative bacterial infections *in vivo*. The bacterial probe photon intensity (PI) was expressed as photon flux in units of photons/s/cm^2^/steradian. To quantify the bacterial probe fluorescence, we defined the regions of interest (ROI) and converted signals to false-color photon-count images using Living Image version 3.0 software (Caliper LS Co., Hopkinton, MA, USA).

We first investigated whether the NIR-fluorescent probe could bind to anionic phospholipids in the bacterial cell membrane to visualize *P. acnes in vitro* (Fig. 2). In small Eppendorf tubes, 50 μl of medium, the NIR-fluorescent control probe, or the NIR-fluorescent bacterial detection probe, was incubated at 37.0 °C under anaerobic conditions for 15 min, and 1 × 10^8 ^CFU of *P. acnes* in 100 μl of medium was then added to each tube. The cell suspension was fixed with 4% paraformaldehyde (PFA), washed twice with phosphate-buffered saline (PBS), and centrifuged at 5000 g for 5 min to collect the precipitated bacteria and decant the supernatant. We next used an IVIS^®^-Lumina CCD optical system to detect the NIR-fluorescent signals from the bacterial probe, which was bound to the bacterial cell membrane.

### Mouse osteomyelitis model

Eighteen adult (12-week-old) male BALB/c mice weighing 20–25 g (Sankyo Labo Service, Shizuoka, Japan) were used. The mice were housed five per cage under specific pathogen-free conditions and maintained on a 12-h light/dark cycle with access to food and water. The mice were fed alfalfa-free food (PicoLab^®^ Select Rodent Diet 50 IF/6F, LabDiet, St. Louis, MO, USA). The mice were anesthetized with an intraperitoneal injection of 50 mg/kg of pentobarbital, and the skin on their left knee was sterilized with povidone iodine as previously described^20^. A skin incision was made over the left knee, and the distal femur was exposed. A drill and 23G needle were used to make a hole at the distal end of the femur^20^, and a 0.5 × 8-mm titanium alloy bar was inserted into the mouse femur along with an inoculation of *P. acnes* (1 × 10^6^ CFU in 1 μl medium) (implant group, N = 12)^46^. The same technique was used for the control group but without the titanium implant (N = 6). The mice were tracked for 6 months. All of the experiments were approved by the Animal Care and Use Committee of Keio University. And, these experiments were carried out in accordance with the approved guidelines.

### Visualization of the inflammation region caused by *P. acnes* infection

To visualize the inflammation region caused by *P. acnes* osteomyelitis, the XenoLight Rediject Inflammation Probe (Chemiluminescent reagent: Summit Pharmaceuticals International Co.) was used. This probe, which detects the myeloperoxidase (MPO) activity of activated neutrophils, enables longitudinal tracking of the MPO level and inflammation status *in vivo*. Using both bacterial probe and inflammation probe, the bacterial growth and inflamed regions can be observed simultaneously in individual animals^21^. Bacterial growth and the inflamed region were detected by FRI and BLI, respectively. Seven days after inoculating *P. acnes* into the femur, the mice were administered 100 μl of the NIR-fluorescent bacterial detection probe by intravenous injection. After 12 h, 150 μl of the chemiluminescent inflammation probe, which enabled neutrophil tracking, was administered intraperitoneally. The mice were anesthetized 1 h later via inhalation of aerosolized 1.5% isoflurane mixed with oxygen, and the bacterial probe fluorescence and inflammation probe luminescence were captured using the trans-illumination feature of the IVIS^®^ Lumina optical-imaging system.

### Optical imaging of *P. acnes* in osteomyelitis over time

To monitor bacterial growth in the mouse osteomyelitis model, 100 μl of the NIR-fluorescent bacterial detection probe was intravenously injected into the tail vein at each time point, and the *P. acnes in vivo* was observed 12 h later using the trans-illumination feature of the IVIS^®^ Lumina optical imaging system (Caliper LS Co.). In this osteomyelitis model, the signal intensity was typically stable at approximately 12 h after injecting the NIR-fluorescent bacterial detection probe; the probe was then gradually washed out and excreted in the urine (data not shown). Mice were placed on their back, and images were captured for 5 s. The bacterial probe PI was expressed as photon flux in units of photons/s/cm^2^/steradian. To quantify the bacterial probe fluorescence, we defined an ROI in the surgical area of the left leg. To normalize the PI, the ROI in the right leg of each image was measured as background. A fluorescence ratio (ROI of the operated left leg/ ROI of the right leg) was calculated for each image. To analyze the time course of infection, the bacterial probe PI in the ROI was sequentially measured on days 1, 3, 7, 14, 28, 56, 84 (3 months), and 168 (6 months) after inoculation.

### Histological Analysis

For histological analysis, femur specimens were collected and analyzed on day 28 after inoculation in both groups. The mice were sacrificed, the femurs were removed and separated from the soft tissues, and the samples were fixed in 4% PFA in 0.1 M PBS and demineralized with ethylenediaminetetraacetic acid. The samples were then embedded in paraffin, cut into 5-μm-thick sections, and Gram stained or hematoxylin and eosin stained.

### *Ex vivo* and scanning electron microscopy analyses

For the *ex vivo* analysis, the NIR-fluorescent bacterial detection probe was injected into the mice 3 or 6 months after *P. acnes* inoculation, and the mice were sacrificed 12 h after injecting the probe. The femurs and implants were removed and separated from the soft tissues. The surface of the retrieved implants was examined using a fluorescence microscope after Cy2 LIVE/DEAD^®^ BacLight staining (Life Technologies Japan). The bacteria were fluorescently labeled with BacLight to monitor the viability of bacterial populations as a function of the membrane integrity of the cell; Cy2 only stains cells with intact membranes^50^.

The surface of implants retrieved 6 months after surgery was analyzed using a SEM (JEOL, JSM-6390LA, Japan). Samples were then fixed for >24 h in 2% PFA and 2% glutaraldehyde at 4 °C, dried, mounted on metal stubs, metal-coated with platinum–palladium using an ion-coater, and observed using a SEM at an accelerating voltage of 5 kV.

### Genetic analysis

The effusions from femurs in the implant and the control groups were examined by anaerobic culture and PCR. Briefly, 6 months after the *P. acnes* inoculation, the left femur was removed and the femoral bone marrow cavity was washed with 10 mL PBS to obtain cells and extracts. The cells and extracts were cultured in Reinforced Clostridial Agar Medium at 37.0 °C under anaerobic conditions for 2 weeks. Samples from cultured colonies and from stocked *P. acnes* (ATCC No. 51277, Strain Designations VPI 9) as a positive control and MSSA (Caliper LS Co.) as a negative control were examined. The 16 S ribosomal RNA (rRNA) of *P. acnes* was amplified by PCR, and DNA from the samples was extracted using the ISOGEN method (Wako Pure Chemical Industries, Osaka, Japan). All PCR cycles began with denaturation for 5 min at 95 °C, followed by 40 cycles of 1 min at 95 °C, 2 min at 54 °C, and 3 min at 72 °C. Samples were then incubated for 5 min at 72 °C for extension. A custom primer, 5′-GGCACACCCATC-TCTGAGCAC-3′ (Sigma Aldrich Japan Co. Tokyo, Japan), was designed to amplify a 587-bp portion of the *P. acnes* 16 S rRNA^51^.

### Statistical Analysis

Differences in bacterial PI between the implant and control groups at each time point were analyzed using Student’s *t-*test. SPSS II software (IBM-SPSS, Tokyo, Japan) was used, and a *P* value < 0.05 was considered significant.

## Additional Information

**How to cite this article**: Shiono, Y. *et al*. Delayed *Propionibacterium acnes* surgical site infections occur only in the presence of an implant. *Sci. Rep.* **6**, 32758; doi: 10.1038/srep32758 (2016).
